# Study of the meiotic segregation of chromosome 7 with a paracentric inversion in spermatosoa of a heterozygous carrier

**DOI:** 10.18699/vjgb-25-71

**Published:** 2025-09

**Authors:** M.M. Antonova, D.A. Yurchenko, Zh.G. Markova, N.V. Shilova

**Affiliations:** Bochkov Research Centre for Medical Genetics, Moscow, Russia; Bochkov Research Centre for Medical Genetics, Moscow, Russia; Bochkov Research Centre for Medical Genetics, Moscow, Russia; Bochkov Research Centre for Medical Genetics, Moscow, Russia

**Keywords:** paracentric inversion, chromosome 7, sperm FISH, meiotic segregation, sperm recombinant chromosomes, парацентрическая инверсия, хромосома 7, FISH сперматозоидов, мейотическая сегрегация, рекомбинантные хромосомы

## Abstract

A paracentric inversion (PAI) is a rare type of balanced intrachromosomal structural rearrangement. Heterozygotes for PAI are usually phenotypically normal, but the presence of the inversion may occasionally lead to synapsis and recombination disruptions during meiosis. PAI can be responsible for the production of recombinant chromosomes and unbalanced gametes. The risks associated with the birth of a child with chromosomal imbalances due to the generation of unbalanced crossover gametes is considered to be low. Nonetheless, viable offspring with intellectual disabilities and/or congenital abnormalities, as well as early miscarriages, stillbirth and infertility in heterozygous carriers of PAI have been described. Paracentric inversions may arise on various chromosomes. PAI with breakpoints on the long arm of chromosome 7 is among the most prevalent ones in humans. To assess the meiotic behavior of abnormal chromosome 7, as well as the empirical risk of producing gametes with recombinant chromosomes, the sperm FISH analysis of a male heterozygous carrier of inv(7)(q11.23q22) was performed. The percentage of recombinant sperms was 0.7 % and chromosomal imbalance was represented as reciprocal breakage products of a dicentric chromosome 7. Notably, spermatozoa with a dicentric chromosome 7 were not observed, which confirms its instability during meiosis I. Meiotic segregation analysis in the heterozygous carrier of inv(7)(q11.23q22) revealed a predominant formation of gametes containing either the inverted or the intact chromosome 7, occurring at frequencies of 52.2 and 47.8 %, respectively. This report is the first study providing a detailed description of meiotic segregation patterns of inv(7)(q11.23q22) by using a sperm FISH approach. Recombinant gamete formation confirms the occurrence of crossing-over within the inversion loop. Consequently, the individual risk of generating gametes (and subsequent zygotes) with chromosome 7 imbalance for this heterozygous carrier remains low.

## Abstract (ru)

Парацентрическая инверсия (ПаИ) – это редкая сбалансированная внутрихромосомная структурная перестройка. Хотя гетерозиготные носители ПаИ обычно не имеют клинически значимых аномалий фенотипа, факт присутствия в кариотипе хромосомы с инвертированным сегментом предопределяет проблемы синапсиса и рекомбинации в мейозе у таких индивидов и приводит к формированию рекомбинантных хромосом с хромосомным дисбалансом. Риск рождения больного ребенка для носителей ПаИ из-за производства несбалансированных гамет в результате мейотической рекомбинации считается низким. Однако были описаны случаи рождения ребенка с нарушением интеллектуального развития и/или пороками развития; случаи спонтанных абортов, бесплодия у носителей из-за классической рекомбинации в инвертированном хромосомном сегменте. ПаИ могут быть сформированы на различных хромосомах. Показано, что у человека одной из частых среди парацентрических инверсий является ПаИ с локализацией точек разрыва в длинном плече хромосомы 7. С целью оценки мейотического поведения хромосомы 7 с парацентрической инверсией в длинном плече и эмпирического риска формирования гамет с рекомбинантными хромосомами проведено молекулярно-цитогенетическое исследование клеток эякулята у мужчины – гетерозиготного носителя ПаИ хромосомы 7 − inv(7)(q11.23q22). Рекомбинантные хромосомы 7 обнаружены с частотой 0.7 % и в гаметах представлены суммарно реципрокными продуктами разрыва дицентрической хромосомы 7. Сперматозоиды с дицентрической хромосомой 7 не обнаружены, что подтверждает факт нестабильности этой хромосомы в мейозе I у носителя данной парацентрической инверсии. Показано, что мейотическая сегрегация у гетерозиготного носителя inv(7)(q11.23q22) проходит с преимущественным формированием гамет с инвертированной и интактной хромосомой 7 с частотой 52.2 и 47.8 % соответственно. Впервые получены сведения о частоте формирования гамет с рекомбинантными хромосомами при мейотической сегрегации inv(7)(q11.23q22), что подтверждает факт наличия кроссинговера в инверсионной петле. Персонализированный риск формирования гамет (зигот) с дисбалансом материала хромосомы 7 у гетерозиготного носителя данной инверсии является низким.

## Introduction

**Fig. 1.**
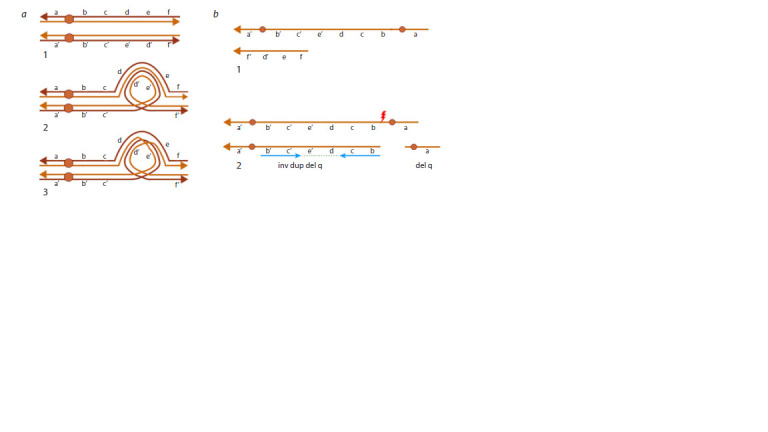
Meiotic segregation of a chromosome with a PAI: a – formation of an inversion loop in meiosis I: homologous chromosomes, the lower one has an inversion (1); formation of an inversion loop (2); crossing-over within the inversion loop (3). b – theoretically possible variants of gametes during meiotic segregation of PAI: dicentric chromosome and acentric fragment (1); break of the dicentric chromosome, forming an inverted duplication with an adjacent terminal deletion (inv dup del) and a chromosome with a terminal deletion of the arm (2). Adapted (Burssed et al., 2022).

Inversion is an intrachromosomal structural rearrangement in which two breaks occur, and the segment lying between the breakpoints rotates 180°. In paracentric inversions (PAI) of chromosomes, both breakpoints are located on the same arm of the same chromosome. Thus, the centromere is not involved in the rearrangement, and the rearranged chromosome consists of an inverted segment and two flanking, distal, non-inverted regions. PAI occurs with a frequency of 0.1–0.5 % (Gardner, Amor, 2018). Most often, PAI is found in chromosomes 1, 3, 5, 6, 7, and 11, with breakpoints localized at 3(p13p25), 6(p12p23), 6(p12p25), 7(q11q22), and 11(q21q23) (Pettenati et al., 1995). Heterozygous carriers of PAI do not exhibit clinically significant phenotypic abnormalities (Madan, 1995; Yang et al., 1997; Muss, Schwanitz, 2007). However, the presence of a chromosome with an inverted segment in the karyotype can lead to problems during meiotic segregation, resulting in the formation of gametes with recombinant chromosomes. This, in turn, may lead to zygotes with chromosomal imbalance and the birth of a child with chromosomal pathology. A key feature of synapsis and recombination in paracentric inversions during the pachytene stage of prophase I is the formation of an inversion loop (Fig. 1a).

**Fig. 2.**
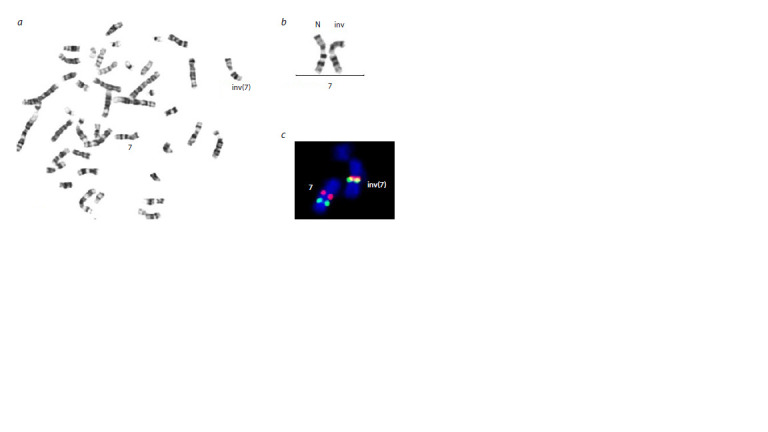
Metaphase plate (a), fragment of the karyogram of the patient with inv(7)(q11.23q22) (b) and the result of hybridization with locus-specific DNA probes on chromosome 7 (c) – the convergence of hybridization signals from locus-specific DNA probes to regions 7q11 (red) and 7q22 (green) in one of the homologues of chromosome 7 indicates the presence of PAI.

Depending on the number of crossovers between a normal chromosome and its PAI homologue, various meiotic segregation outcomes are possible. If crossing-over occurs outside the inversion loop, no recombinant chromosomes will form. A single crossover within the inversion loop can lead to the formation of a recombinant dicentric chromosome and an acentric fragment (Fig. 1b-1) (Phelan et al., 1993; Anton et al., 2005). Cells containing an acentric fragment undergo apoptosis. The dicentric chromosome is unstable and may rupture during anaphase of meiosis I, resulting in gametes with abnormal chromosomes: one with an inverted duplication and an adjacent terminal deletion (inv dup del) and the other with a terminal deletion of the chromosome arm (Feldman et al., 1993; Mitchell et al., 1994) (Fig. 1b-2). The empirical risk of gametes with recombinant chromosomes can be assessed using FISH analysis of ejaculate cells (Bhatt et al., 2009; Balasar, Acar, 2020). In cases of classical segregation leading to an unstable dicentric chromosome, commercially available DNA probes targeting the centromeric and subtelomeric regions of the chromosome with PAI are sufficient for analysis.

Reports on meiotic segregation in inversion carriers show wide variability in the frequency of recombinant gametes, ranging from 0 to 38 % (Morel et al., 2007; Anton et al., 2005; Bhatt et al., 2009). This variability influences the reproductive outcomes for couples where one partner carries an inversion. For male heterozygous carriers of PAI, determining the frequency of abnormal gametes allows for personalized risk assessment of having a child with chromosomal imbalance and improves medical and genetic counseling for the family.

The aim of our study was to evaluate meiotic segregation of chromosome 7 with paracentric inversion in ejaculate cells and determine the frequency of gametes with recombinant chromosomes

## Materials and methods

The patient was a healthy 41-year-old man without clinical phenotypic abnormalities, enrolled in an assisted reproductive technology (ART) program for male infertility. Samples of peripheral venous blood and ejaculate were collected for analysis.

Cytogenetic study was performed on cultured peripheral blood lymphocytes according to a standard protocol (Cytogenetic Methods…, 2009). GTG-banding (550 bands) revealed the karyotype 46,XY,inv(7)(q11.23q22).

The inverted segment size relative to the q arm and the total length of chromosome 7 were calculated as 27.4 and 16.8 %, respectively.

Preparations from spermatozoa were obtained in accordance with a previously developed protocol (Tarlycheva et al., 2021).

FISH analysis of spermatozoa was performed using DNA probes on the centromeric region of chromosome7 (SE 7 (D7Z1), SpBlue), subtelomeric region of the long arm of chromosome 7 (Subtel 7q, SpRed), subtelomeric region of the long arm of chromosome 2 (Subtel 2q, SpGreen) as a control of ploidy and hybridization efficiency (Leica, Kreatech, Germany) according to the protocol of the manufacturing company. FISH analysis of peripheral blood lymphocytes was performed using locus-specific DNA probes on chromosome 7 labeled with various fluorochromes: ELN (7q11) (SpO)/7q22 (SpG) (Leica, Kreatech, Germany).

Hybridization signals were analyzed using an Axio Imager M.1 epifluorescence microscope (Carl Zeiss, Germany) and Isis software (MetaSystems, Germany).

## Results

FISH analysis of peripheral blood lymphocytes confirmed PAI in the patient (Fig. 2).

**Fig. 3.**
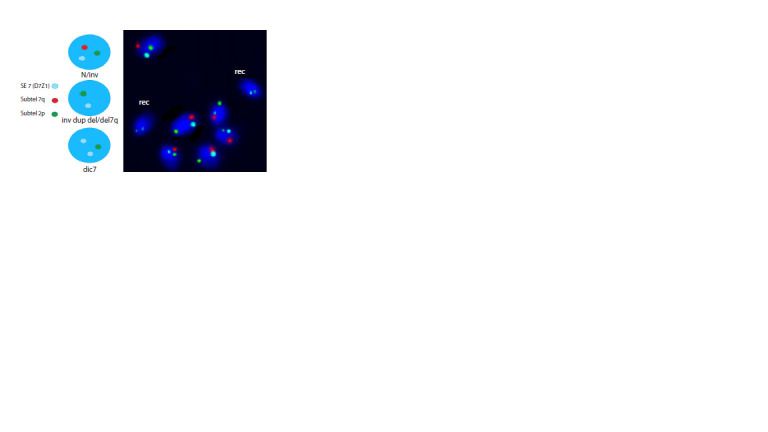
Hybridization patterns expected in gametes due to crossingover in the inversion loop in a male carrier of inv(7)(q11q22) and the result of FISH analysis performed on preparations from the ejaculate of a heterozygous inv(7)(q11q22) carrier to estimate the frequency of gametes with recombinant chromosome 7.

To evaluate the frequency of gametes with recombinant and non-recombinant (normal and inverted) chromosome 7, FISH analysis of the patient’s ejaculate cells was performed using a combination of DNA probes targeting the subtelomeric region of the long arm and the centromeric region of chromosome 7, as well as the subtelomeric region of the short arm of chromosome 2. In gametes with non-recombinant chromosomes, one blue, one red, and one green hybridization signal should be observed. In gametes with recombinant chromosomes – inv dup del(7q) or del(7q) – only one blue (from the centromeric region of chromosome 7) and one green (control) hybridization signal will be present, while the red hybridization signal will be absent, as all such chromosomes exhibit a terminal deletion of the long arm of chromosome 7. Meanwhile, gametes with a recombinant dicentric chromosome can be identified by the presence of two blue hybridization signals (corresponding to the centromeric region of chromosome 7) and one green control signal (Fig. 3).

The results of the frequency analysis of gametes with non-recombinant (normal and balanced) and recombinant chromosome 7 are presented in Table 1. During the analysis of 6,116 ejaculate cells, recombinant chromosome 7 was detected at a frequency of 0.7 %, and in mature germ cells (gametes), it was represented exclusively by reciprocal products of the breakage of a dicentric chromosome 7. Spermatozoa carrying the recombinant dicentric chromosome were not detected, confirming the instability of this chromosome during meiosis I in the carrier of this paracentric inversion.

**Table 1.**
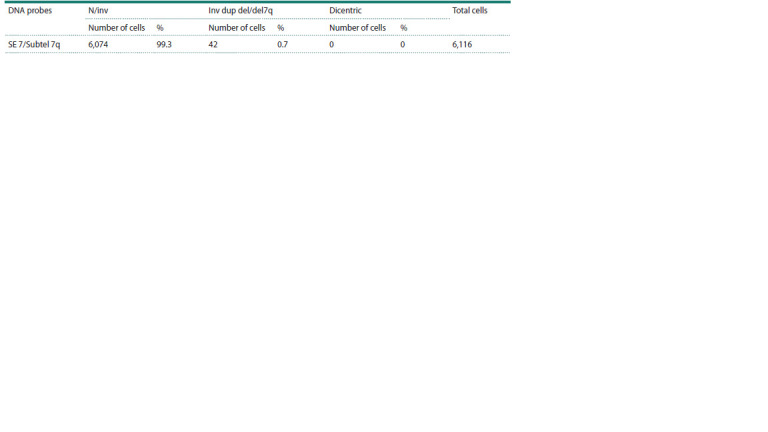
Frequency of gametes with non-recombinant and recombinant chromosome 7

**Fig. 4.**
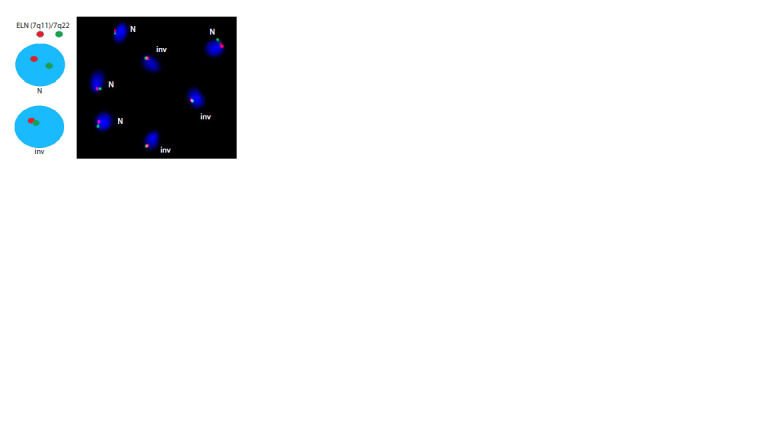
Hybridization patterns enabling evaluation of gamete types in the absence of recombination within the inversion loop in a male carrier of inv(7)(q11.23q22), and the results of FISH analysis on ejaculate preparations from a heterozygous inv(7)(q11.23q22) carrier for assessing the frequency of gametes with intact and inverted chromosome 7.

To assess the frequency of gametes with intact and inverted chromosome 7, FISH analysis was performed using a combination of DNA probes targeting the q11 (red hybridization signal) and q22 (green hybridization signal) regions of chromosome 7. The distance between the hybridization signals allowed for the determination of whether chromosome 7 was intact or inverted. In the case of an inversion, the red and green hybridization signals appeared closer together. The hybridization results and possible signal patterns are presented in Figure 4.

The results of the analysis of the frequency of gametes with intact and inverted chromosome 7 are presented in Table 2. A total of 3,250 cells were analyzed, with the frequency of cells carrying inverted and intact chromosome 7 being 52.2 and 47.8 %, respectively

**Table 2.**
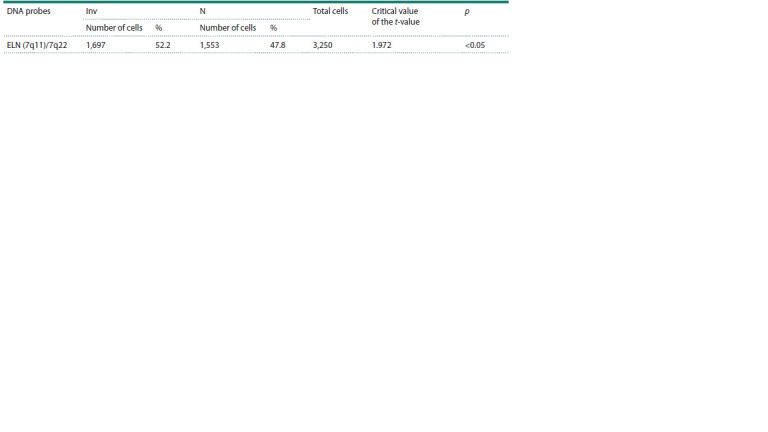
Frequency of gametes with intact and inverted chromosome 7

## Discussion

Constitutional chromosomal abnormalities are among the known genetic factors contributing to male infertility, increased risk of miscarriage, and the birth of children with developmental disorders. Paracentric inversions (PAIs) can not only disrupt meiosis and spermatogenesis but also lead to the formation of mature gametes with chromosomal imbalance due to the generation of recombinant chromosomes during male gametogenesis. The classic meiotic segregation scenario for PAIs involves crossing-over within the inversion loop, followed by the formation of a dicentric chromosome, its subsequent breakage, and the production of gametes carrying inv dup del and deleted chromosomes.

Multiple factors influence the formation of the inversion loop, including the size of the inverted segment. The risk of generating gametes with recombinant chromosomes depends on the likelihood of meiotic crossing-over occurring within the inversion loop. If the inversion is small, the probability of crossing-over within the inverted segment is low, as the number of crossover events appears to be proportional to chromosome length. Studies on the meiotic segregation of pericentric inversions have demonstrated that when the inverted segment constitutes <30 % of the chromosome length, recombinant gametes are not formed. If the inverted segment spans 30–50 % of the chromosome length, the frequency of recombinant gametes is <5 %, increasing to 20.5 % when the inverted segment exceeds 50 % (Morel et al., 2007). A positive correlation between the size of the inverted segment and the frequency of recombinant gametes has also been observed in the limited studies on the meiotic behavior of PAIs. For instance, an analysis of meiotic segregation patterns in blastocysts during preimplantation genetic testing of couples carrying PAIs revealed that the frequency of blastocysts with recombinant chromosomes increased with the size of the inverted segment, ranging from complete absence (when the inversion was <37.5 % of the chromosome length) to 12 % (for larger inversions) (Xie et al., 2019). Our previous research also demonstrated that in a heterozygous carrier of a polymorphic PAI in the short arm of chromosome 8 (with the inverted segment constituting 3.2 % of the chromosome length), the frequency of recombinant gametes was 0.03 % (Yurchenko et al., 2022)

Since only one chromosome arm is involved in the paracentric inversion and findings indicate that synapsis initiates distally on both arms in metacentric and submetacentric chromosomes but involves only one arm in acrocentric chromosomes (Brown et al., 1998), it was proposed to modify the evaluation criteria for PAIs. Instead of calculating the size of the inverted segment relative to the entire chromosome, it should be calculated relative to the length of the arm containing the inversion. S. Bhatt et al. demonstrated that when the PAI size is less than 50 % of the corresponding chromosome arm length, the percentage of recombinant spermatozoa ranges from 0 to 3.72 %, increasing to 10 % or more when the PAI exceeds 50 % of the arm length (Bhatt et al., 2014). In the present case of a heterozygous carrier of inv(7)(q11.23q22), where the inverted segment constitutes 16.8 % of chromosome 7 length and 27.4 % of its q-arm, the frequency of recombinant gametes was 0.7 %. These findings support the established correlation between the size of the inverted segment and recombination frequency in PAIs.

Limited studies on male gametogenesis in PAI carriers have reported an absence of recombinant chromosomes during meiotic segregation of inv(7)(q11q22) (Bhatt et al., 2009, 2014). The authors refer to an original study (Martin, 1986) in which meiotic segregation analysis was performed on pronuclear chromosomes obtained via in vitro penetration of spermatozoa from an inv(7)(q11q22) carrier into golden hamster (Mesocricetus auratus) oocytes. After analyzing 94 metaphase spreads, the authors concluded that no recombinant chromosome 7 was present (Martin et al., 1986).In our analysis assessing the frequency of recombinant chromosome 7, hybridization patterns were examined in over 6,000 ejaculate cells. This allowed us to obtain reliable evidence of recombinant chromosomes in a heterozygous inv(7)(q11.23q22) carrier, contradicting previous findings

The frequency of gametes with inverted chromosome 7 was statistically significantly different (p < 0.05) from that of gametes with intact chromosome 7. Thus, we suggest that heterozygous inv(7)(q11.23q22) carriers exhibit a preferential tendency to produce gametes with inverted chromosome 7 during meiotic segregation. However, drawing definitive conclusions is challenging due to the potential for random signal proximity, which could introduce systematic bias and overestimate the frequency of gametes with inverted chromosome 7.

## Conclusion

A key aspect of genetic counseling for families carrying chromosomal rearrangements is assessing the risk of having children with chromosomal abnormalities caused by pathological segregation patterns during gametogenesis in the parent carrying the rearrangement. Determining the degree of genetic risk, along with the potential medical and social consequences of the anticipated chromosomal pathology, enables the development of personalized preventive strategies to avoid the birth of an affected child. FISH analysis of ejaculate cells is a specific method for studying the meiotic behavior of chromosomal abnormalities, including paracentric inversions. By identifying an effective combination of DNA probes for molecular cytogenetic analysis of male gametogenesis, it becomes possible to investigate segregation patterns and evaluate recombination events occurring during meiosis in carriers of chromosomal abnormalities. The assessment of the risk of having a child with chromosomal imbalance directly depends on understanding the frequency of recombinant gamete formation.This study demonstrates that meiotic segregation of the paracentric inversion inv(7)(q11.23q22) in the long arm of chromosome 7 predominantly results in gametes carrying either an intact or inverted chromosome 7. For the first time, data on the frequency of recombinant gamete formation during meiotic segregation of inv(7)(q11q22) have been obtained, confirming the occurrence of crossing-over within the inversion loop. The personalized risk of producing gametes (or zygotes) with chromosomal imbalance in a heterozygous carrier of inv(7)(q11q22) is 0.7 %, which is considered low.

## Conflict of interest

The authors declare no conflict of interest.
